# Advanced paternal age directly impacts mouse embryonic placental imprinting

**DOI:** 10.1371/journal.pone.0229904

**Published:** 2020-03-06

**Authors:** Michelle M. Denomme, Jason C. Parks, Blair R. McCallie, Nathan I. McCubbin, William B. Schoolcraft, Mandy G. Katz-Jaffe

**Affiliations:** 1 Fertility Labs of Colorado, Lone Tree, CO, United States of America; 2 Colorado Center for Reproductive Medicine, Lone Tree, CO, United States of America; University of Missouri Columbia, UNITED STATES

## Abstract

The placental epigenome plays a critical role in regulating mammalian growth and development. Alterations to placental methylation, often observed at imprinted genes, can lead to adverse pregnancy complications such as intrauterine growth restriction and preterm birth. Similar associations have been observed in offspring derived from advanced paternal age fathers. As parental age at time of conception continues to rise, the impact of advanced paternal age on these reproductive outcomes is a growing concern, but limited information is available on the molecular mechanisms affected *in utero*. This longitudinal murine research study thus investigated the impact of paternal aging on genomic imprinting in viable F1 embryonic portions of the placentas derived from the same paternal males when they were young (4–6 months) and when they aged (11–15 months). The use of a controlled outbred mouse model enabled analysis of offspring throughout the natural lifetime of the same paternal males and excluded confounding factors like female age or infertility. Firstly, paternal age significantly impacted embryonic placental weight, fetal weight and length. Targeted bisulfite sequencing was utilized to examine imprinted methylation at the *Kcnq1ot1* imprinting control region, with significant hypermethylation observed upon natural paternal aging. Quantitative real-time PCR assessed imprinted gene expression levels at various imprinting clusters, resulting in transcript level alterations attributable to advanced paternal age. In summary, our results demonstrate a paternal age effect with dysregulation at numerous imprinted loci, providing a mechanism for future adverse placental and offspring health conditions.

## Introduction

As couples delay childbearing to later stages in life, the impact of increasing parental age on reproductive outcomes has become a significant concern [1]. Advanced paternal age (APA) is associated with various pregnancy complications including increased risks for placental abruption, miscarriage, premature birth and low birth weight [2–4]. Long-term effects from delayed fatherhood have also been reported in mice, including reproductive fitness and longevity [5] as well as postnatal development and behavioral traits in offspring [6]. However, limited information is available on the direct influence of APA on the molecular mechanisms driving embryonic development *in utero*.

Our preceding murine study showed an adverse effect of APA on *in vivo* and *in vitro* reproductive outcomes commencing at paternal midlife [7]. One potential underlying mechanism involves perturbations in epigenetic modifications, such as DNA methylation, which regulate gene expression without alterations to the DNA sequence. Gametogenesis and embryogenesis are important stages for the establishment and maintenance of epigenetic marks. It is thus hypothesized that epigenetic errors during spermatogenesis can occur over time as males age. In fact, age-related methylation errors have already been identified in human sperm [8, 9] as well as mouse sperm [10, 11]. Evidence has shown a critical role for the paternal epigenome in embryo development, and so temporal alterations to the sperm epigenetic landscape as fathers age may be inherited by progeny and subsequently affect offspring development.

The placental epigenome plays a key role in regulating embryonic growth and development. Alterations in embryonic placental DNA methylation, frequently occurring at imprinted genes, have been associated with adverse pregnancy complications including preterm birth, intrauterine growth restriction and preeclampsia [12, 13]. Genomic imprinting is a phenomenon that leads to monoallelic gene expression based on parental-origin [14]. Imprinted domains often comprise of multiple genes under coordinated epigenetic control. The *Kcnq1ot1* imprinting cluster consists of a maternally-methylated imprinting control region (ICR) with paternally-expressed *Kcnq1ot1* non-coding RNA and numerous maternally-expressed protein-coding genes [15]. Importantly, a large proportion of imprinted genes are involved in growth, development and metabolism, and play critical roles in both the placenta and the brain.

The aim of this longitudinal research study was to investigate the impact of paternal aging in a controlled outbred murine model on the imprinted methylation of the *Kcnq1ot1* ICR and gene expression at various imprinting clusters in viable embryonic placentas. The use of a mouse model enabled analysis throughout the natural lifetime of the paternal males and excluded confounding factors like female age or infertility. Our results demonstrate a paternal age effect on embryonic placental and fetal weight, likely due to widespread dysregulation of imprinted genes restricted to the paternal allele, providing a mechanism for future adverse offspring health conditions.

## Materials and methods

### Ethics statement

This study conducted in a privately-owned corporate setting followed national ethical guidelines for humane animal treatment and complied with relevant legislation. All experimental designs including studies involving animals were approved by an internal Research Committee, adhering to a high standard of animal care in strict accordance with the recommendations in the Guide for the Care and Use of Laboratory Animals (8th Edition). Euthanasia was performed by cervical dislocation, and all efforts were made to minimize suffering.

### Animals and male aging

Eight 6–8 week old outbred CF1 male mice (Charles River) with proven fertility were randomly selected for this study. Throughout the study, only young fertile 6–8 week old outbred CF1 female mice were selected for matings.

### Placenta collection

Young male CF1 mice with proven fertility (“paternal males”) were mated routinely from 4–15 months of age with superovulated young CF1 females (6–8 week old). Before mating, females underwent an ovarian superovulation protocol as described previously [16], by an injection of 5 IU PMSG (Sigma) followed 48 hours later by 5 IU hCG (Sigma). The pregnant females were sacrificed each month at E16 of fetal development and the F1 embryonic portions of the placentas (“embryonic placentas”; n = 8 males, 96 placentas total) were carefully dissected by excising the superficial portion of the chorion/chorionic plate and stored at -80°C; this tissue has been shown to contain no maternal cells [17]. Embryonic placental weight (g), fetal weight (g) and crown-rump length (mm) were also measured and recorded. Student’s t-test was performed, with differences considered to be significant at p<0.05. DNA and RNA were concurrently isolated from embryonic placentas derived from the same males in their youth (4–6 months) and aged (11–15 months) (All-in-One Purification Kit, Norgen) and stored at -80°C for further processing.

### Targeted bisulfite sequencing

F1 embryonic placenta samples derived from the same males (n = 8) in their youth (48 placentas) and aged (48 placentas) were analyzed for targeted DNA methylation analysis as previously described [18]. Briefly, DNA isolation of individual embryonic placenta samples using the QiaAmp DNA Mini kit (Qiagen) was followed by bisulfite mutagenesis using the EZ DNA Methylation-Direct kit (Zymo Research). PCR amplification involved the addition of 4 ng converted DNA directly to Hot Start Ready-To-Go (RTG) (GE Healthcare) PCR beads that each contained 0.4 μM *Kcnq1ot1* primers, 1 μL of 240 ng/mL transfer RNA and water up to 25 μL, with 25 μL mineral oil overlay. Methylation primers and PCR parameters are outlined in the supplementary data (S1A Table). PCR products were gel extracted, ligated into the pGEM-T EASY vector system (Promega), and transformed into Z-competent DHα *Escherichia coli* cells (Zymo Research). Following colony PCR amplification, 30 μL of individual clone samples were sent for sequencing at Bio Basic Inc. (Markham, ON, Canada). Approximately 26–28 clones were sequenced for each of the 96 samples. Methylation patterns were determined using two online software programs (BISMA and QUMA). Identical clones with the same CpG methylation and unconverted cytosines were considered to be representative of one individual DNA strand, and thus were included only once. Total DNA methylation for each gene was calculated as a percentage of the total number of methylated CpGs divided by the total number of CpG dinucleotides. Two-way ANOVA statistical assessments were used to examine significance for methylation between paternal age groups and individual paternal males for 4–6 vs. 11–15 months (n = 8) and aged subgroups 4–6 vs. 11–12 months (n = 5), and 4–6 vs. 14–15 months (n = 3), while a one-way ANOVA test was used to evaluate significance between the young group and the two aged subgroups (4–6 vs. 11–12 vs. 14–15 months). Percent methylation differences with p<0.05 were considered to be statistically significant.

### Quantitative real-time PCR

Analysis of transcript abundance on isolated RNA from the same individual embryonic placenta samples (96 placentas from 8 males) was performed as previously described [19]. Briefly, RNase-free DNase I (Qiagen) treated samples were reverse transcribed using the High Capacity Reverse Transcription cDNA kit (Thermo Fisher Scientific). Quantitative real-time PCR was performed with Power SYBR Green PCR Master Mix (ThermoFisher Scientific) on the ABI 7300 Real Time PCR System. Quantification of nineteen imprinted genes within five imprinting clusters were calculated relative to a constant internal housekeeping gene, *Ppia*, and the embryonic placenta samples derived from aged males (11–15 months, and subdivided into 11–12 months and 14–15 months) were analyzed relative to those derived from when the same males were in their youth (4–6 months). Expression primers and PCR parameters are outlined in the supplementary data (S1B Table). The Relative Expression Software Tool (REST 2009; Qiagen) was used for mRNA gene expression analysis. REST uses a mathematical model for gene expression which is based on the PCR efficiencies and the mean crossing point deviation between sample and control group and tested for significance relative to a housekeeping gene by a pairwise fixed reallocation randomization test [20]. Gene expression fold differences with p<0.05 were considered to be statistically significant. Furthermore, two-way ANOVA statistical analysis was performed on deltaCt values to evaluate the effects of both paternal age as well as the paternal male individual for 4–6 vs. 11–15 months (n = 8), with p<0.05 considered statistically significant.

## Results

### Reproduction and fetal development

Outbred males (n = 8) with proven fertility were mated every consecutive month with superovulated young outbred females throughout their natural lifetime. For purposes of this study, paired litters of offspring from the same individual males were assessed from young paternal males (4–6 months) and the same paternal males when aged (11–15 months). A secondary subdivision of the aged samples (11–12 months vs. 14–15 months) was executed to further characterize the timing of paternal age effects. The greatest age interval for a single male was 10 months, with the smallest age interval being 6 months (Table 1).

**Table 1:** Paternal age at time of offspring embryonic placenta collection.

	Young paternal males (months)	Same paternal males when aged (months)	Interval (months)
Male1	5	15	10
Male2	4	14	10
Male3	6	14	8
Male4	4	11	7
Male5	4	11	7
Male6	6	12	6
Male7	5	11	6
Male8	5	11	6

Pregnancies conceived by the males when aged resulted in significantly smaller embryonic placentas (aged: 0.111g vs. young: 0.149g; p<0.0001), as well as significantly smaller fetuses in both weight (aged: 0.379g vs. young: 0.432g; p<0.05) and length (aged: 13.0mm vs. young: 13.7mm; p<0.05) compared to when the same males were in their youth (Table 2). The fetus: placenta weight ratio was significantly higher for offspring conceived by the males when aged (aged: 3.45 vs. young: 3.05; p<0.05) compared to when the same males were in their youth, indicating placental efficiency is increasingly compromised in the offspring following paternal aging. No statistical differences were observed for the three tissues upon aged subdivision of paternal males at 11–12 months and 14–15 months. The sex of the fetuses was not examined and is a limitation to the study.

**Table 2:** Average offspring development results from the same males in their youth and aged.

	Offspring from young paternal males (paternal age 4–6 months; n = 48)	Offspring from same males when aged (paternal age 11–15 months; n = 48)	P-Value (4–6 vs 11–15 months)	Aged Subdivision (paternal age 11–12 months; n = 30)	Aged Subdivision (paternal age 14–15 months; n = 18)	P-Value (11–12 vs 14–15 months)
Embryonic placental weight (g)	0.149 ± 0.04	0.111 ± 0.02	<0.0001	0.111 ± 0.02	0.111 ± 0.03	n.s.
Fetal weight (g)	0.432 ± 0.08	0.379 ± 0.11	0.0095	0.394 ± 0.11	0.355 ± 0.11	n.s.
Crown-rump length (mm)	13.7 ± 1.3	13.0 ± 1.6	0.0178	13.2 ± 1.6	12.6 ± 1.5	n.s.
Fetus:Placenta weight ratio (#)	3.05	3.45	0.0147	3.28	3.56	n.s.
Successful mating frequency (%)	54.2%	28.1%	0.0494	37.5%	18.8%	n.s.
Fetuses per paternal male (#)	14.8 ± 7.2	18.9 ± 10.3	n.s.	19.8 ± 10.2	17.0 ± 12.5	n.s.

While all eight males successfully mated in their youth, the frequency of mating activity significantly declined for the paternal males when aged (28% successful matings in months 11–15; p<0.05). This decline was exacerbated by increasing paternal age, with only 2/8 males resulting in a successful pregnancy at 14 months of age and only 1/8 resulting in a successful pregnancy at 15 months of age. Nevertheless, upon successful mating the litter size was unchanged between the paternal males in their youth (14.8 ± 7.2 fetuses) and when they aged (18.9 ± 10.3 fetuses; p = n.s.). Analysis of placental and fetal weight for litters of comparable sizes demonstrated an equivalent reduction for offspring conceived by the males when aged compared to their youth (p<0.05).

### DNA methylation at the *Kcnq1ot1* imprinting control region

**Fig 1:**
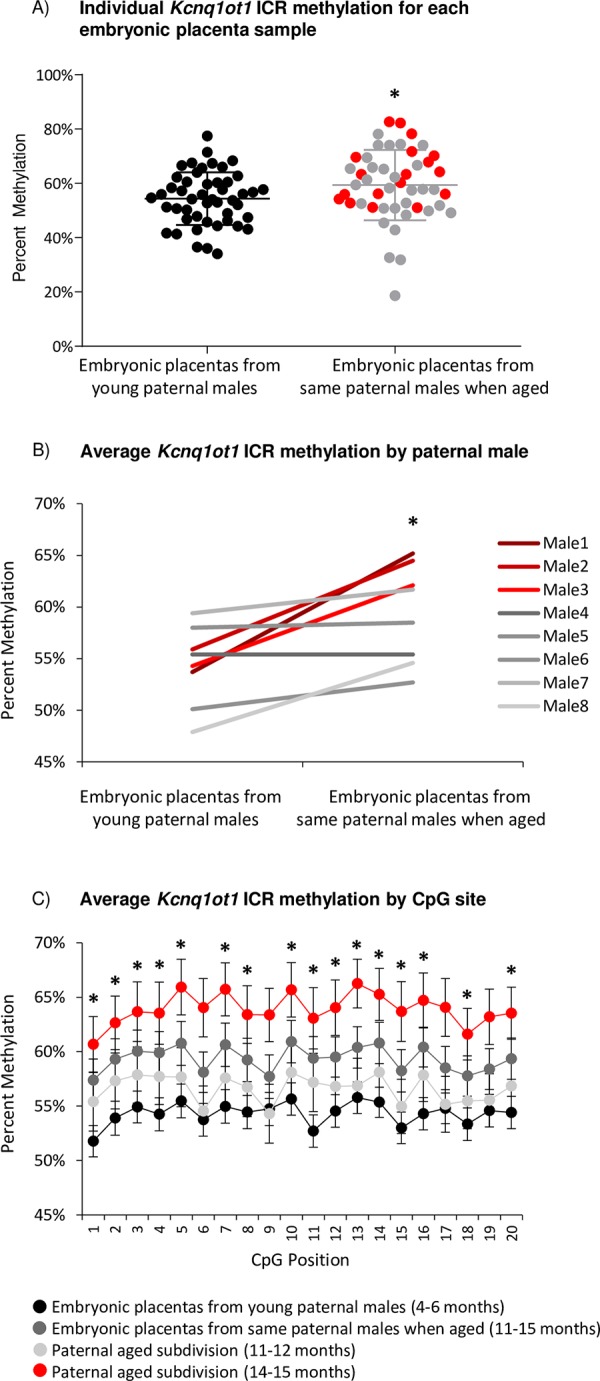
A) Percent methylation at the *Kcnq1ot1* ICR in all individual embryonic placentas derived from young paternal males at 4–6 months (black dots; n = 48) compared to the same paternal males when aged (n = 48); subdivided into 11–12 months (grey dots; n = 30) and 14–15 months (red dots; n = 18). Bolded middle line denotes the mean; error bars represent standard deviation. B) Average percent methylation at the *Kcnq1ot1* ICR for embryonic placentas from each of the eight young paternal males (n = 6/male) and the same paternal males when aged (n = 6/male), subdivided into 11–12 months (grey lines; n = 5 males) and 14–15 months (red lines; n = 3 males). C) Average percent methylation for 20 CpG sites within the amplified *Kcnq1ot1* ICR from embryonic placentas derived from young paternal males (black dots; n = 48) compared to the same paternal males when aged (dark grey dots; n = 48), subdivided into 11–12 months (light grey dots; n = 30) and 14–15 months (red dots; n = 18). Error bars represent standard error of the mean. Statistical significance of p<0.05 is denoted by a star.

Several imprinted genes required for normal placentation are regulated by the *Kcnq1ot1* ICR and the non-coding RNA *Kcnq1ot1* specifically expressed on the paternal allele during development [21]. Results from targeted bisulfite sequencing analyses at the *Kcnq1ot1* ICR revealed a significant DNA methylation difference in embryonic placentas based on the paternal age of individual males. A small but statistically significant increase in imprinted methylation was observed in the paternal aged group (59.3% average) compared to when the same males were in their youth (54.3% average; p<0.05; Fig 1A) due to paternal aging, with no statistical effect based on the individual paternal males (p = n.s.). Average percent methylation increase after aging ranged from 0% to 11.5% per paternal male (Fig 1B, S1 Fig). This range correlated appropriately with age, such that the greatest methylation differences were observed in the embryonic placentas derived from the males with largest age intervals and oldest males (14–15 months). Of the twenty CpG sites analyzed within the *Kcnq1ot1* ICR, sixteen were found to be statistically significant between the two groups (p<0.05; Fig 1C).

To further characterize the timing of the methylation aberrations, the embryonic placentas from the paternal males when aged were subdivided into 11–12 months (n = 5 males, 30 placentas) and 14–15 months (n = 3 males, 18 placentas). Methylation at the *Kcnq1ot1* ICR was significantly increased only in the placentas derived from the 14–15 month aged males (63.9% average) compared to the 11–12 month aged males (56.6% average) and to the same males in their youth (4–6 months; 54.3% average; p<0.05), again with no statistical effect based on the individual paternal males (p = n.s.), demonstrating the targeted increase in imprinted DNA methylation over time, specifically as the males aged past 12 months.

### Gene expression for the *Kcnq1ot1* imprinting cluster

**Fig 2:**
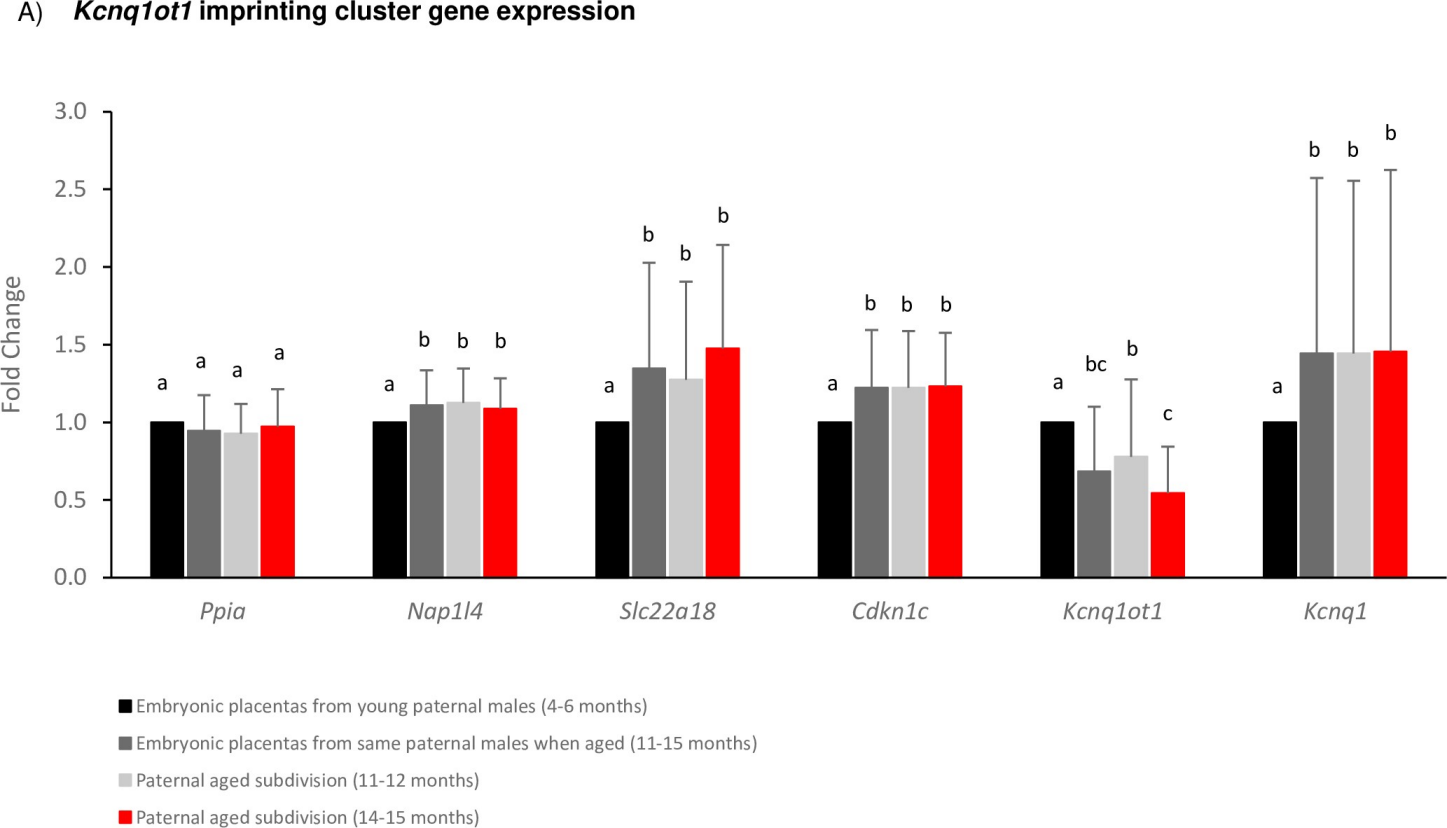
A) Expression results for imprinted genes within the *Kcnq1ot1* imprinting cluster for embryonic placentas derived from the same males in their youth (4–6 months, black bars; n = 48) and aged (11–15 months, dark grey bars; n = 48), subdivided into 11–12 months (light grey bars; n = 30) and 14–15 months (red bars; n = 18). Alphabetic letters indicate statistical significance of p<0.05 between groups.

Results from imprinted gene expression analyses revealed corresponding differences between embryonic placentas based on paternal age. Five genes analyzed within the maternally-methylated *Kcnq1ot1* imprinting cluster were all observed to be differentially expressed in the paternal aged group compared to when the same males were in their youth. A significant decrease in transcript abundance was observed due to paternal age for the paternally-expressed *Kcnq1ot1* non-coding RNA and a significant increase in expression for the paternally-silenced protein-coding genes *Nap1l4*, *Slc22a18*, *Cdkn1c*, and *Kcnq1* (p<0.05; Fig 2), with the individual paternal males resulting in a statistical effect at *Kcnq1ot1* and *Slc22a18* (p<0.05). Upon subdivision of the aged group, the paternally-expressed *Kcnq1ot1* non-coding RNA was found to be further significantly downregulated in the embryonic placentas derived from the paternal males when aged to 14–15 months (p<0.05). This pattern of expression combined with the observed hypermethylation is indicative of imprinting dysregulation restricted to the paternal allele based on paternal aging.

### Gene expression for additional imprinting clusters

**Fig 3:**
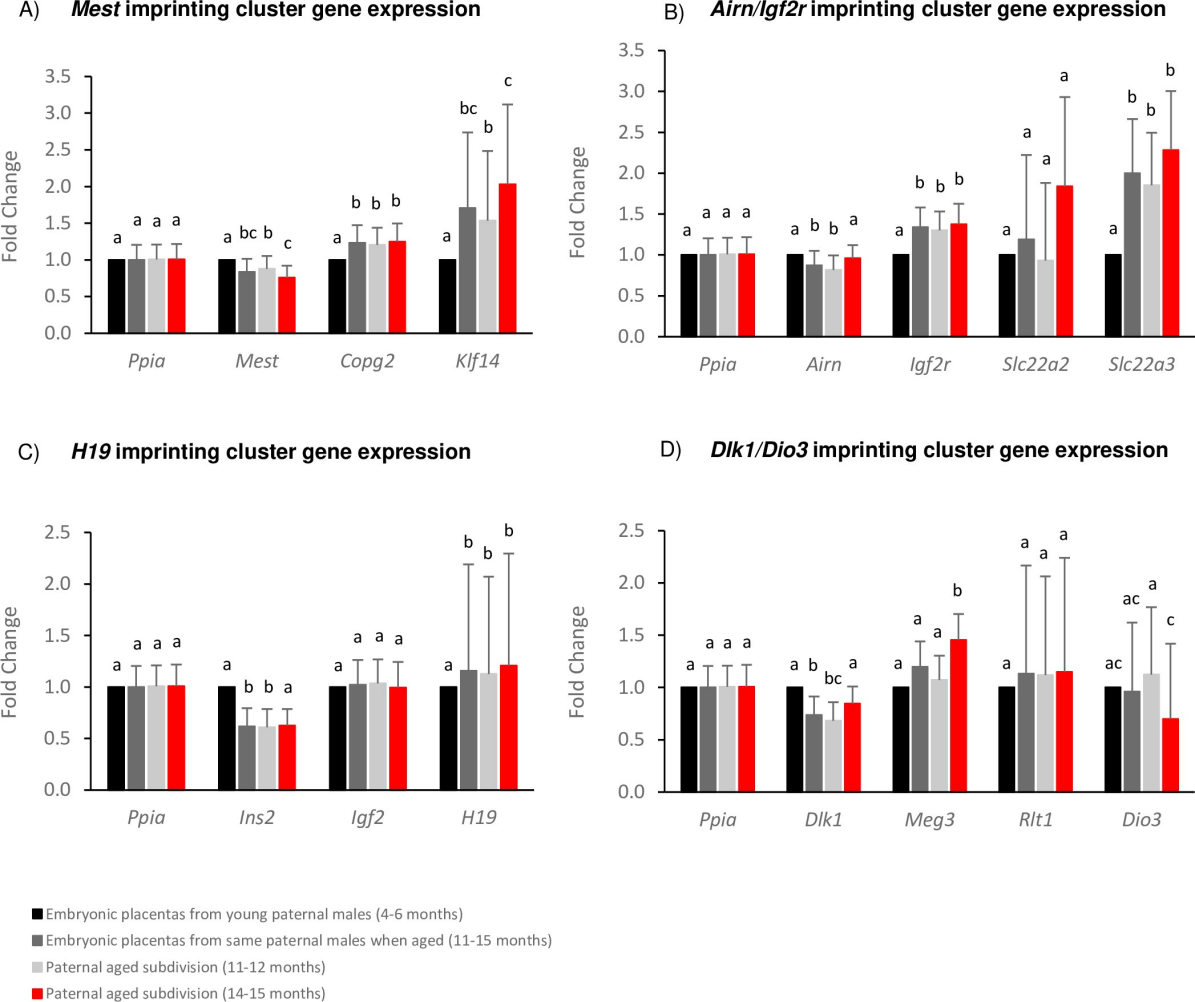
Expression results for imprinted genes within the A) *Mest* B) *Airn/Igf2r* C) *H19* D) *Dlk1/Dio3* imprinting clusters for embryonic placentas derived from the same males in their youth (4–6 months, black bars; n = 48) and aged (11–15 months, dark grey bars; n = 48), subdivided into 11–12 months (light grey bars; n = 30) and 14–15 months (red bars; n = 18). Alphabetic letters indicate statistical significance of p<0.05 between groups.

Four additional imprinting clusters were analyzed for gene expression alterations in the same embryonic placentas. For the maternally-methylated *Mest* imprinted domain, *Mest* transcript abundance was significantly decreased, while *Copg2* and *Klf14* were significantly increased with paternal age (p<0.05; Fig 3A). Similarly, in the maternally-methylated *Airn/Igf2r* imprinted domain, the non-coding RNA *Airn* was significantly decreased, with significantly increased expression of *Igf2r* and *Slc22a3* (p<0.05; Fig 3B). In the paternally-methylated *H19* imprinted domain, the non-coding RNA *H19* transcript abundance was significantly increased, and *Ins2* was significantly decreased with paternal aging (p<0.05; Fig 3C). Finally, in the paternally-methylated *Dlk1/Dio3* imprinted domain, *Dlk1* expression was significantly decreased (p<0.05; Fig 3D). A statistical effect was also observed based on the individual paternal males at *Mest*, *Copg2* and *Slc22a3* (p<0.05). Upon subdivision of the aged group, *Mest*, *Klf14* and *Dio3* were significantly dysregulated in the embryonic placentas derived from the paternal males when aged to 14–15 months compared to those aged 11–12 months. *Airn*, *Ins2* and *Dlk1* reached statistical significance only in those aged 11–12 months, while *Meg3* was found to be significantly increased only in those aged 14–15 months; which did not reach significance when combined with the 11–12 months cohort. The subtle and varying effect of APA, combined with the reduced sample sizes when subdivided, may contribute to the variable statistical differences observed at certain imprinted genes. Significant decreases in gene expression for paternally-expressed imprinted genes, and corresponding significant increases in gene expression for paternally-silenced imprinted genes are indicative of imprinting dysregulation restricted to the paternal allele due to paternal aging.

## Discussion

While the genome is largely reprogrammed during gametogenesis and early embryogenesis, the epigenetic status at imprinted domains must be actively maintained, and thus are highly susceptible to epigenetic perturbations. In our longitudinal research study, paternal aging during the natural lifetime of male mice was associated with significant dysregulation to both ICR DNA methylation and imprinted gene expression in embryonic placentas of their offspring.

The *Kcnq1ot1* ICR and the paternally-expressed non-coding RNA *Kcnq1ot1* regulate several imprinted genes required for normal placentation during development [21]. *Kcnq1ot1* ICR methylation in eight sets of paired embryonic placenta samples showed overall hypermethylation upon advanced paternal age (APA) compared to when the same males were in their youth. While the average methylation was situated near expected for imprinting loci, the range of methylation between placenta samples was considerable even when the sperm contribution was from the males in their youth, highlighting variability between individuals (both F0 and F1). A larger placental methylation range was, nevertheless, observed when the same males aged during their natural lifetimes. Most embryonic placentas had increased methylation, but markedly decreased methylation was also observed in a few select samples, indicative of overall epigenetic perturbations in either direction by advanced paternal age. Hypermethylation of the *Kcnq1ot1* ICR was exacerbated in the embryonic placentas derived from the paternal males when they reached 14–15 months of age, demonstrating the targeted increase in imprinted DNA methylation over time, specifically as the males aged past 12 months. The gain of methylation in the majority of samples is presumed to come from the unmethylated paternal allele, as the maternal allele is hypermethylated. This aberrant gain of methylation at a gametic imprinting control region may have originated in the APA sperm and been directly inherited by the offspring, or it may be attributable to a paternal factor(s) compromised in the aging sperm environment leading to inappropriate imprinting methylation maintenance in the trophectoderm of the embryo. The biological significance of the small methylation shift is unknown, though subtle epigenetic changes have been associated with complex disease phenotypes [22], and may result in a cumulative effect on placentation and embryonic developmental capacity. As paternal aging is a subtle and varying effect, not every pregnancy from an APA father will be impacted. Indeed, a threshold of methylation alterations may be required to predispose placental dysfunction. As allelic identity was not feasible, overrepresentation of the maternal allele is also a possibility; a substantial number of samples were analyzed for both young and aged groups in order to overcome this technical limitation.

The subtle, yet statistically significant shift in DNA methylation at the *Kcnq1ot1* ICR based on advanced paternal age lead to subsequent transcription changes for five imprinted genes within the cluster, observed in both subdivisions of the paternally aged group. In particular, the embryonic placentas derived from males that aged to 14–15 months had the largest methylation increase, and this correlated with the largest *Kcnq1ot1* expression decrease, statistically. These alterations may be due to disrupted transcription factor binding. Within the amplified *Kcnq1ot1* ICR region, various binding sites exist (MatInspector by Genomatix), many with direct binding motifs overlapping at least one CpG site. These transcription factors have potential to be impacted by compacted chromatin from increased methylation, including those that have been shown to be critical in genomic imprinting regulation. For example, the Kruppel-like zinc finger protein ZFP57 binds methylated DNA and is necessary for maintaining the methylation memory at imprinting control regions during replication in early mouse embryos [23]. Binding of ZFP57 to the aberrantly methylated *Kcnq1ot1* ICR paternal allele would result in a more maternal-like phenotype by means of recruiting additional repressive factors. Evidence of this is the aberrant decrease in *Kcnq1ot1* gene expression, and the resulting aberrant increase in the surrounding imprinted genes, analogous to what is observed on the maternal allele. The loss of *Kcnq1ot1* transcript expression is presumed to occur on the paternal allele, as the maternal allele is innately silenced, supporting a paternally-inherited effect at the *Kcnq1ot1* imprinted domain from advanced paternal age.

A large proportion of imprinted genes are involved in growth, development and metabolism, and play important roles in both the placenta and the brain. Paternally-expressed genes often promote growth enhancement while maternally-expressed genes are involved in growth suppression. Loss of mouse paternally-expressed *Mest* causes placental growth restriction, while loss of maternally-expressed *Igf2r* or *Cdkn1c* results in placental enlargement [24]. In the embryonic placentas derived from males in their old age, significantly decreased expression was observed in paternally-expressed genes (*Kcnq1ot1*, *Mest*, *Airn*, *Ins2*, *Dlk1*) and aberrant expression in paternally-silenced genes (*Kcnq1*, *Cdkn1c*, *Slc22a18*, *Nap1l4*, *Copg2*, *Klf14*, *Igf2r*, *Slc22a3*, *H19*) compared to when the same males were in their youth. These results in combination with significantly reduced embryonic placental weight is indication of imprinting dysregulation restricted to the paternally-inherited allele due to paternal aging, despite the origin (sperm or oocyte) of methylation for the gametic imprinting control region. The main role of the placenta is the nutrition of the fetus, thus influencing the significant reduction in fetal weight and crown-rump length also observed in the offspring derived from the paternal males when they aged.

The majority of imprinted genes have been described as belonging to imprinted gene networks (IGN) involved in the control of embryonic growth, that are co-regulated during development [25, 26]. For example, *H19*, with dysregulated gene expression in our study, is a master regulator of an IGN impacting expression of other imprinted genes including several that were also dysregulated in our study (*Cdkn1c*, *Dlk1*, *Igf2r* [27, 28], and *Mest*, *Meg3* [28]). Interestingly, various reports of multi-locus imprinting disturbances (MLID) exist in individuals with human imprinting disorders, having epigenetic errors in auxiliary imprinted regions in addition to the disease-associated locus [29–31]. The statistically significant alterations to fourteen imprinted gene transcripts at five imprinting clusters in our study support both IGN and MLID models and provide a translational consequence to the subtle epigenetic dysregulation observed.

It is hypothesized that epimutations in the male gamete may subsequently be generationally transmitted. Age-related imprinting errors have been identified in mouse spermatozoa [10], as well as genome-wide sperm methylation alterations, transferring some methylation abnormalities to offspring and impacting offspring behavior [11]. Furthermore, placental imprinting perturbations have been shown to arise from other external insults to the paternal germline, including paternal folic acid exposure [32] and paternal obesity [33], presumably impacting imprinting on the paternal allele. In terms of aging, DNA methylation alterations were also observed at the *Kcnq1ot1* promoter in the brain of offspring of older fathers compared with the offspring of younger fathers [34]. Our study further argues this generational epigenetic consequence to paternal aging.

In conclusion, the present study examined the contribution of male aging throughout their natural lifetime to genomic imprinting regulation in offspring embryonic placentas, with results demonstrating ICR epigenetic dysregulation, a widespread effect on imprinted expression, and a combined reduction in placental weight, fetal weight, crown-rump length and successful mating frequency. A key strength to the study is the longitudinal feature, enabling age comparisons during the natural lifetime of the same males without confounding factors including female aging or infertility. Imprinted genes play critical roles in growth, development and metabolism, and are highly essential to placental function. Understanding the causal relationship between sperm aging and placental genomic imprinting regulation is critical as developed countries continue to delay childbearing. If these epigenetic changes related to paternal aging are translational, they could be responsible for placental dysfunction as well as adverse pregnancy outcomes and childhood health conditions observed in the human population.

## Supporting information

S1 Fig DNA methylation results for each embryonic placenta for the eight paternal males when young (n = 6/male) and aged (n = 6/male). A) Male1 (ID 61), B) Male2 (ID 18), C) Male3 (ID 116), D) Male4 (ID 83), E) Male5 (ID 90), F) Male6 (ID 112), G) Male7 (ID 53), H) Male8 (ID 81). Each group of circles represents one embryonic placenta sample, with the sample name indicated in the top left (Y = young, A = aged), and percent methylation indicated in the top right. Each row represents one DNA strand. Filled circles represent methylated CpG dinucleotides and unfilled circles represent unmethylated CpGs.
(PDF)

S1 Table A) Primer and PCR parameters for targeted bisulfite sequencing. B) Primer and PCR parameters for targeted qRT-PCR.
(XLSX)
