# Supplementary material for: Advanced paternal age directly impacts mouse embryonic placental imprinting
Source: PLoS One. 2020 Mar 6;15(3):e0229904. doi: 10.1371/journal.pone.0229904 (PMC7059926; doi:10.1371/journal.pone.0229904)

Supplementary Figure S1

A) Male1 (Mouse #61)

Embryonic placentas from paternal youth (5 months)

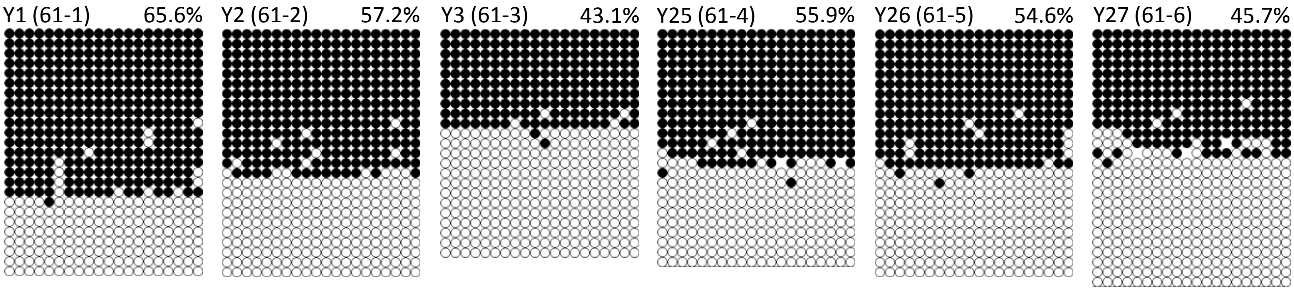

Embryonic placentas from paternal aged (15 months)

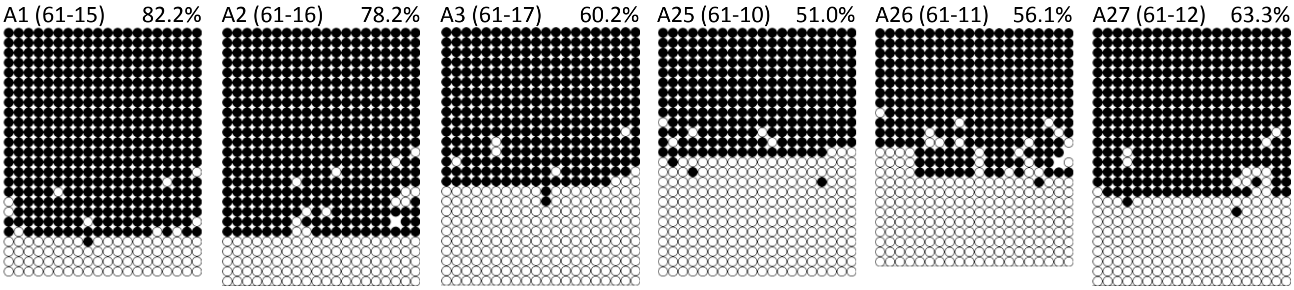

B) Male2 (Mouse #18)

Embryonic placentas from paternal youth (4 months)

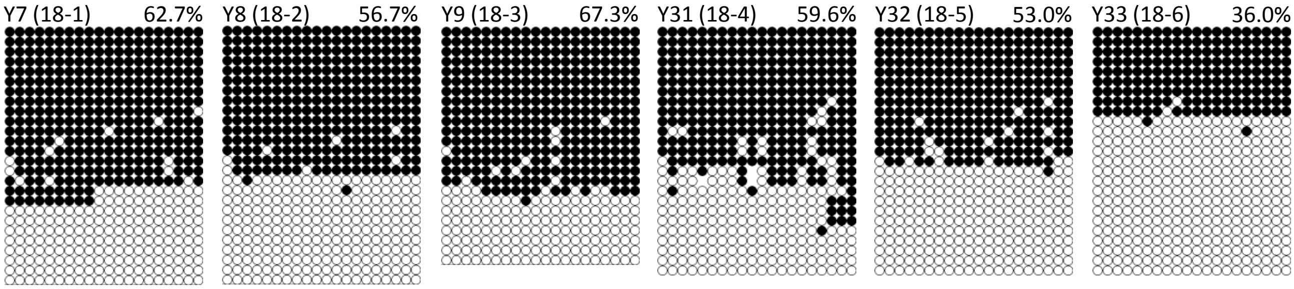

Embryonic placentas from paternal aged (14 months)

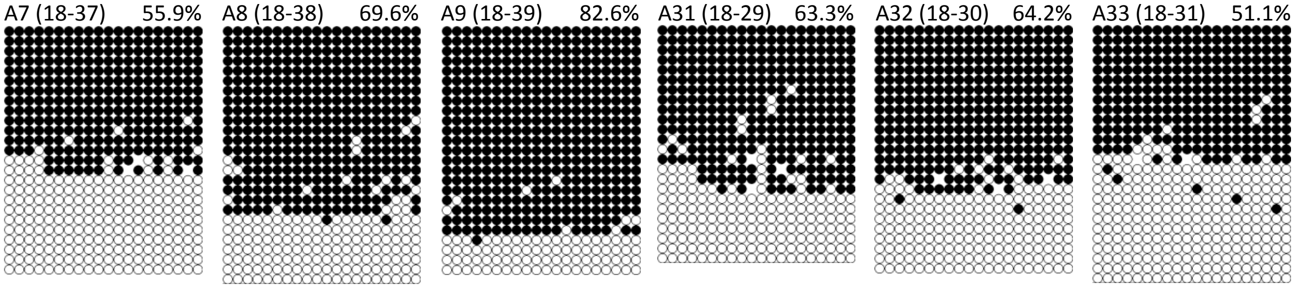

Supplementary Figure S1

C) Male3 (Mouse #116)

Embryonic placentas from paternal youth (6 months)

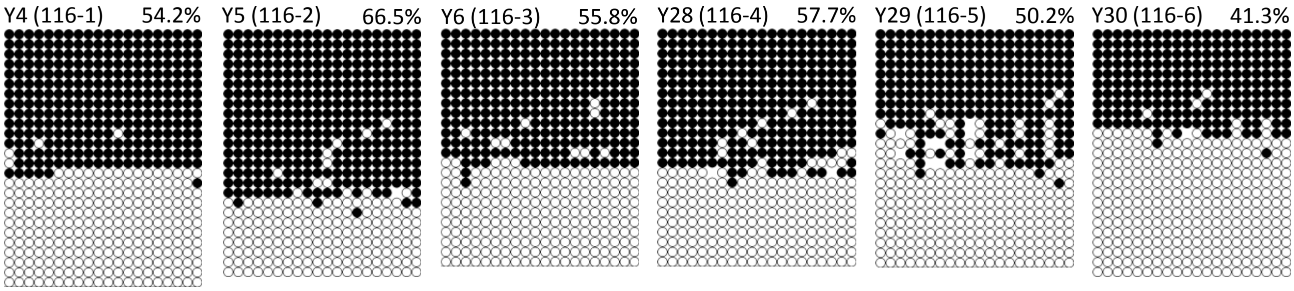

Embryonic placentas from paternal aged (14 months)

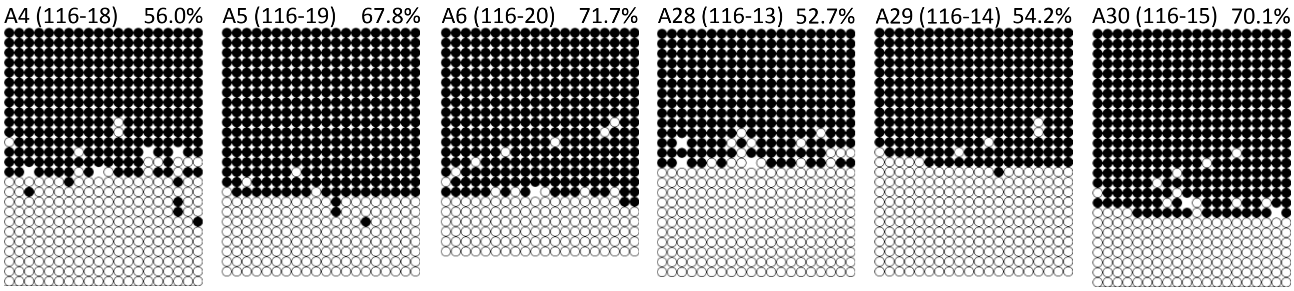

D) Male4 (Mouse #83)

Embryonic placentas from paternal youth (4 months)

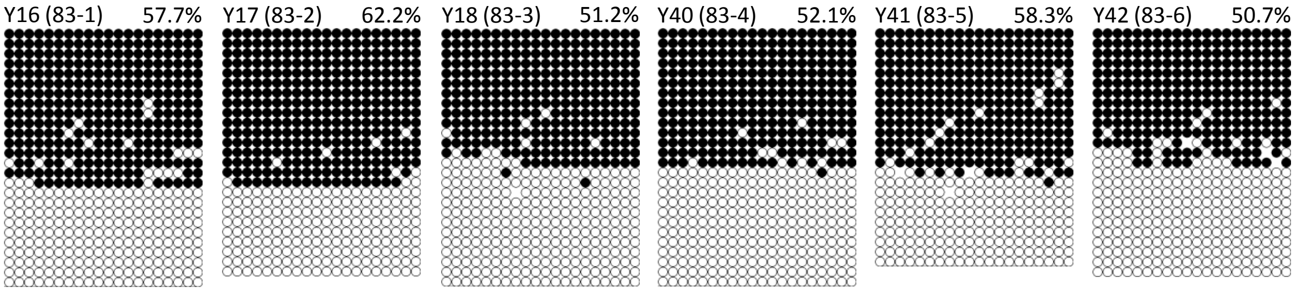

Embryonic placentas from paternal aged (11 months)

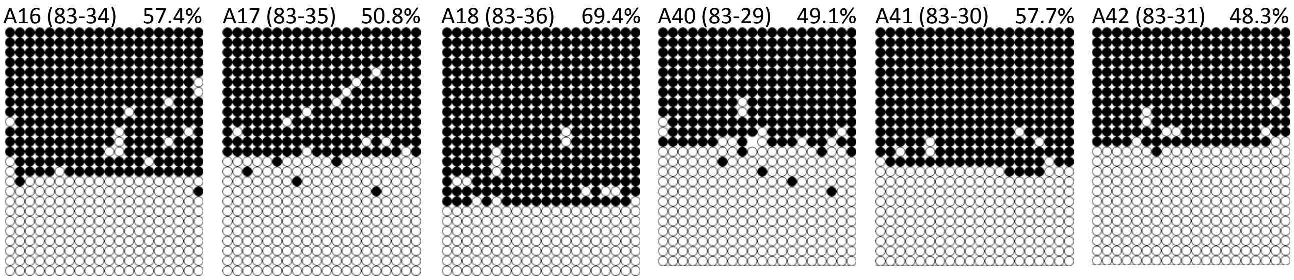

Supplementary Figure S1

E) Male5 (Mouse #90)

Embryonic placentas from paternal youth (4 months)

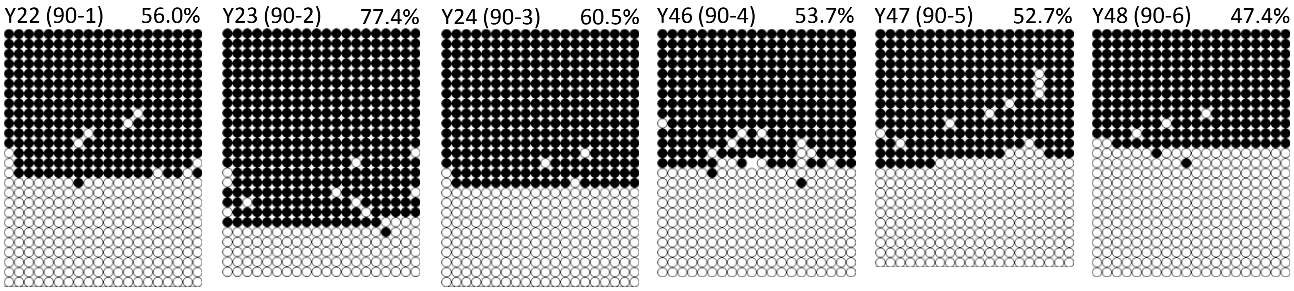

Embryonic placentas from paternal aged (11 months)

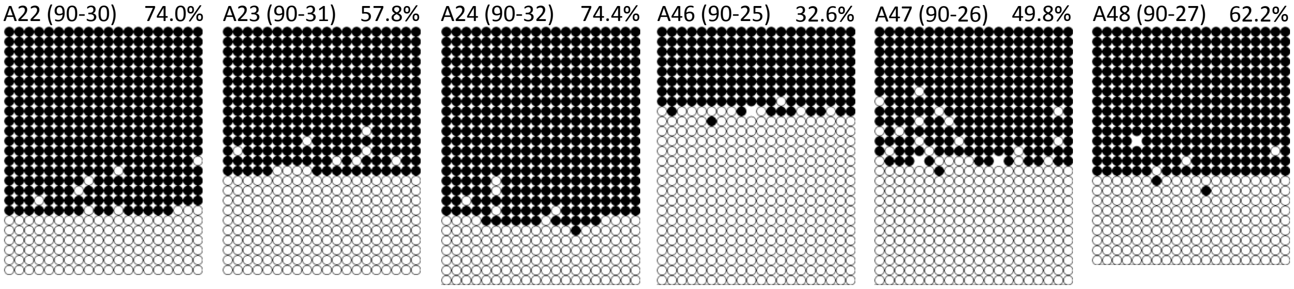

F) Male6 (Mouse #112)

Embryonic placentas from paternal youth (6 months)

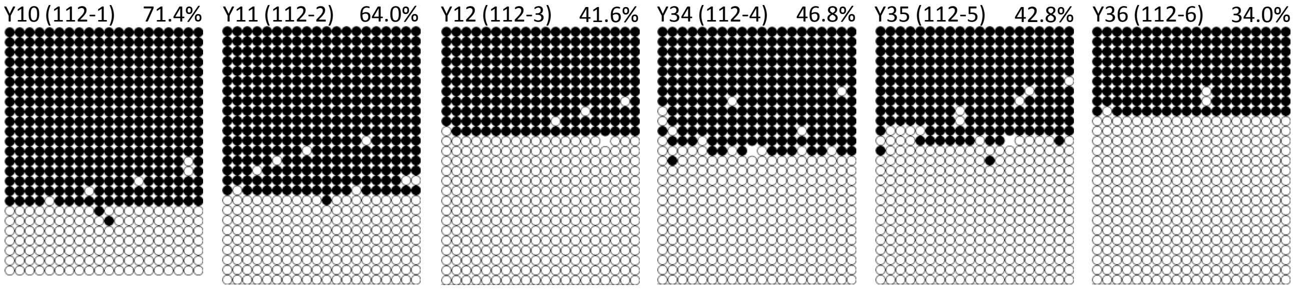

Embryonic placentas from paternal aged (12 months)

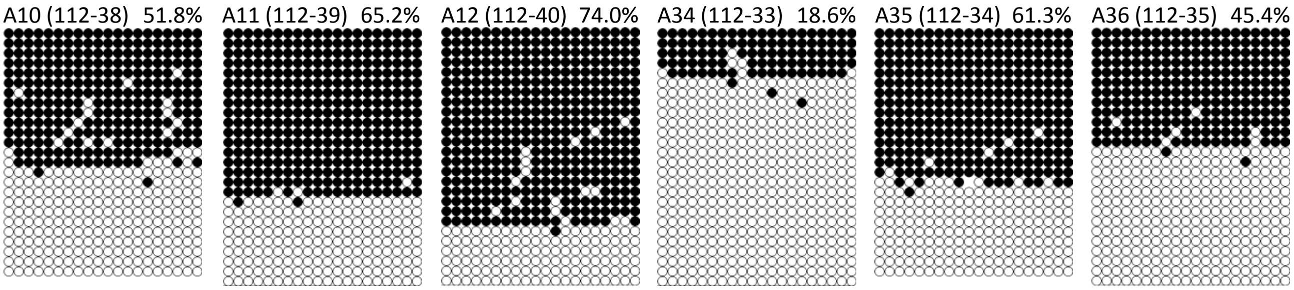

Supplementary Figure S1

G) Male7 (Mouse #53)

Embryonic placentas from paternal youth (5 months)

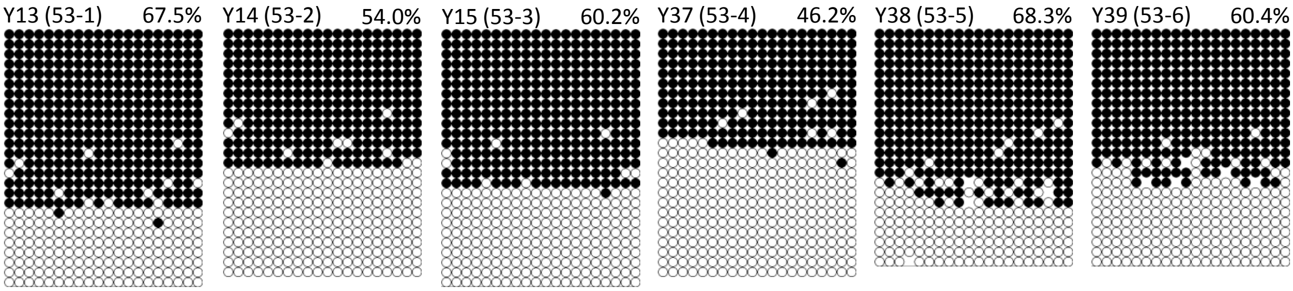

Embryonic placentas from paternal aged (11 months)

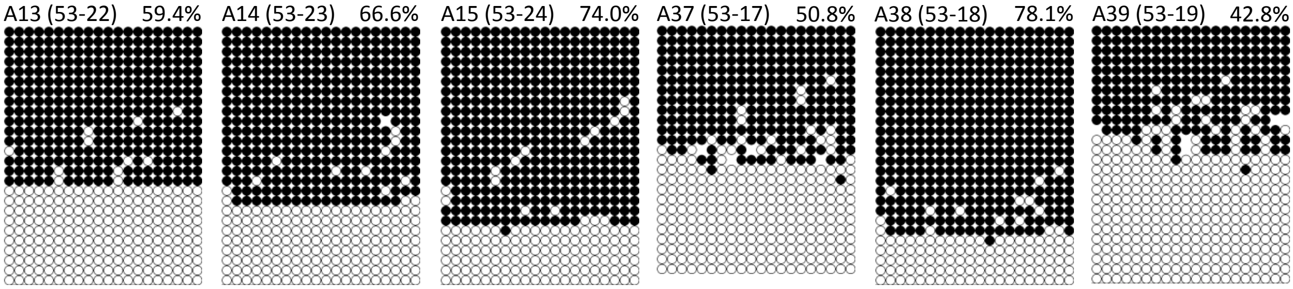

H) Male8 (Mouse #81)

Embryonic placentas from paternal youth (5 months)

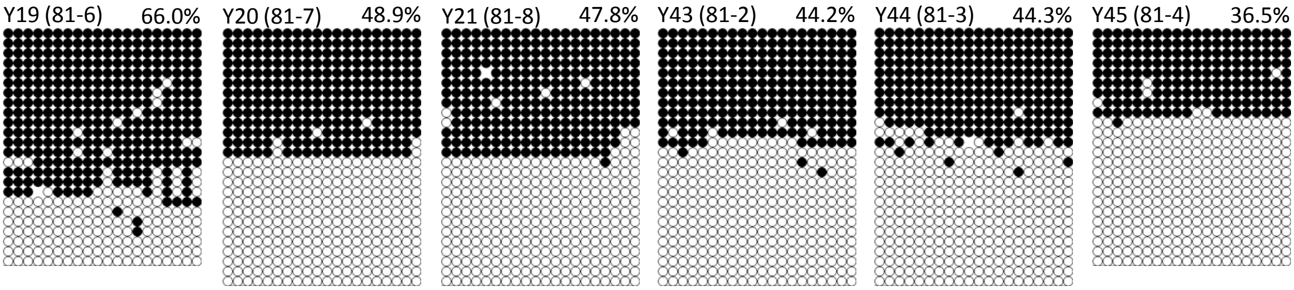

Embryonic placentas from paternal aged (11 months)

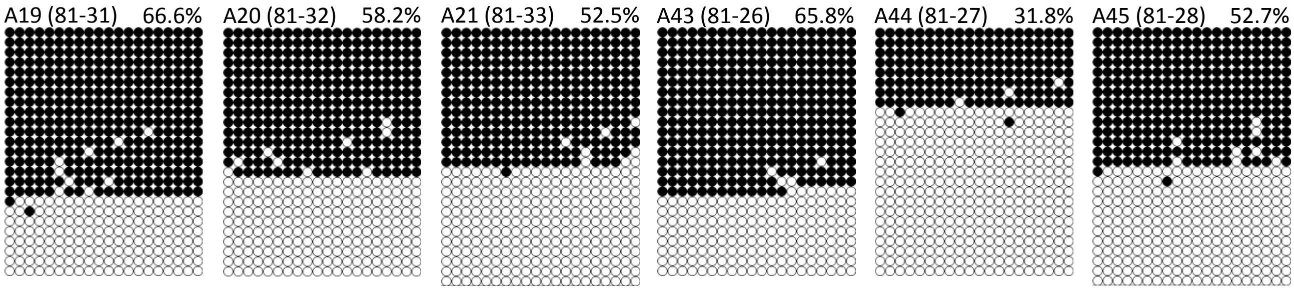

Supplement: S1 Fig — A) Male1 (ID 61), B) Male2 (ID 18), C) Male3 (ID 116), D) Male4 (ID 83), E) Male5 (ID 90), F) Male6 (ID 112), G) Male7 (ID 53), H) Male8 (ID 81). Each group of circles represents one embryonic placenta sample, with the sample name indicated in the top left (Y = young, A = aged), and percent methylation indicated in the top right. Each row represents one DNA strand. Filled circles represent methylated CpG dinucleotides and unfilled circles represent unmethylated CpGs. (PDF) [file pone.0229904.s001.pdf]
